# Multi-nodal basin drainage in lower limb melanoma

**DOI:** 10.1016/j.jpra.2023.11.009

**Published:** 2023-11-24

**Authors:** Adam Hague, Bethany Platford, Saif Ur Rehman, Andrea J. Howes, Philip Brackley

**Affiliations:** Department of Plastic Surgery, Whiston Hospital, Prescot, L35 5DR, United Kingdom

**Keywords:** Melanoma, Sentinel lymph node, Lower limb, Metastases

## Abstract

Lymph node status is an important factor that influences outcomes in melanoma. Whilst certain anatomical areas have multiple-nodal basin drainage, limb melanomas are thought to have more predictable lymphatic drainage patterns, with lower limb melanomas reliably draining to the corresponding ipsilateral inguinal lymph node basin with occasional popliteal drainage. Here we share our unique experience of a patient with a lower limb melanoma demonstrating sentinel lymph nodes, and subsequent metastatic spread, in both the ipsilateral and contralateral inguinal lymph node basins, highlighting an important learning point with respect to our clinical examination of melanoma patients.

Dear Editor,

An important factor that influences outcomes in melanoma is lymph node status.^1^ Since its inception for cutaneous melanoma in 1992, sentinel lymph node biopsy (SLNB) has played a pivotal role in determining this.^2^ However, prior to performing a SLNB and during a patient's subsequent follow up, accurate clinical examination of relevant lymph node basins is vital. It is well known that certain anatomical areas have multiple-nodal basin drainage, namely the trunk and head and neck regions.^3^ In direct contrast to this, limb melanomas are thought to have more predictable lymphatic drainage patterns, with lower limb melanomas reliably draining to the corresponding ipsilateral inguinal lymph node basin with occasional popliteal drainage.^3^ Here, however, we share our unique experience of a patient with a lower limb melanoma demonstrating sentinel lymph nodes and subsequent metastatic spread in both the ipsilateral and contralateral inguinal lymph node basins, highlighting an important learning point with respect to our clinical examination of melanoma patients.

A 62-year-old female was referred to our service with a 3.6 mm Breslow thickness pT3b melanoma on the inferior portion her left anterior thigh. This was diagnosed and completely excised through an excision biopsy. After the relevant counselling she decided to proceed with a SLNB alongside the wide local excision under general anaesthetic. There was no history of previous operations to the lower limb or pelvis and no palpable lymphadenopathy. Pre-operative lymphoscintigraphy was performed on the day of surgery which identified four sentinel lymph nodes in total. Three were in the ipsilateral groin, however, one was also identified in the contralateral groin (Figure 1). Due to this unusual finding further imaging using SPECT-CT was performed to ensure the initial images seen were not produced by artefact on clothing or skin. This confirmed the presence of a contralateral sentinel node (Figure 2). As there remained no palpable lymphadenopathy, the wide local excision and SLNB of the above nodes was subsequently performed and the patient was discharged the same day. The resulting histology demonstrated no residual melanoma in the wide local excision, however, two out of three nodes from the left groin, as well as the single node from the right groin were positive for metastatic melanoma. 3.8 mm parenchymal and 1 mm subcapsular deposits were observed in the ipsilateral nodes whilst a 4 mm parenchymal and 2 mm subcapsular deposit was seen in the contralateral node. Extracapsular spread was present in all three. A subsequent staging CT scan demonstrated multiple nodules within the right lung, as well as enlarged left external iliac lymph nodes. To further characterise these areas a PET CT was performed which identified the lung nodules as likely benign, however, the iliac lymph nodes appeared suspicious for malignancy. After discussion with the patient she opted for medical management and was subsequently referred to oncology. An interval scan has been arranged in order to monitor her lung nodules.

**Figure 1:**
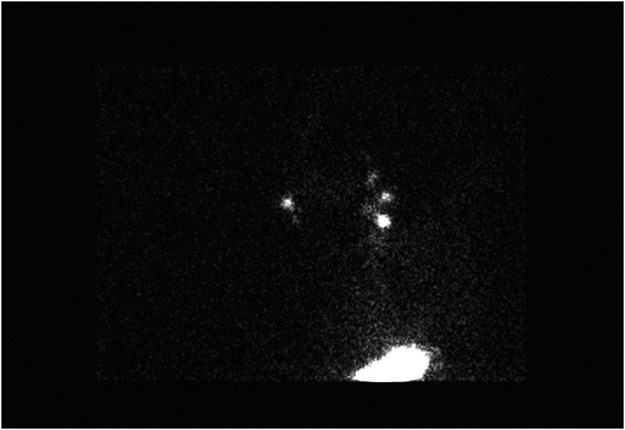
Lymphoscintigraphy showing four inguinal sentinel nodes (three ipsilateral and one contralateral).

**Figure 2:**
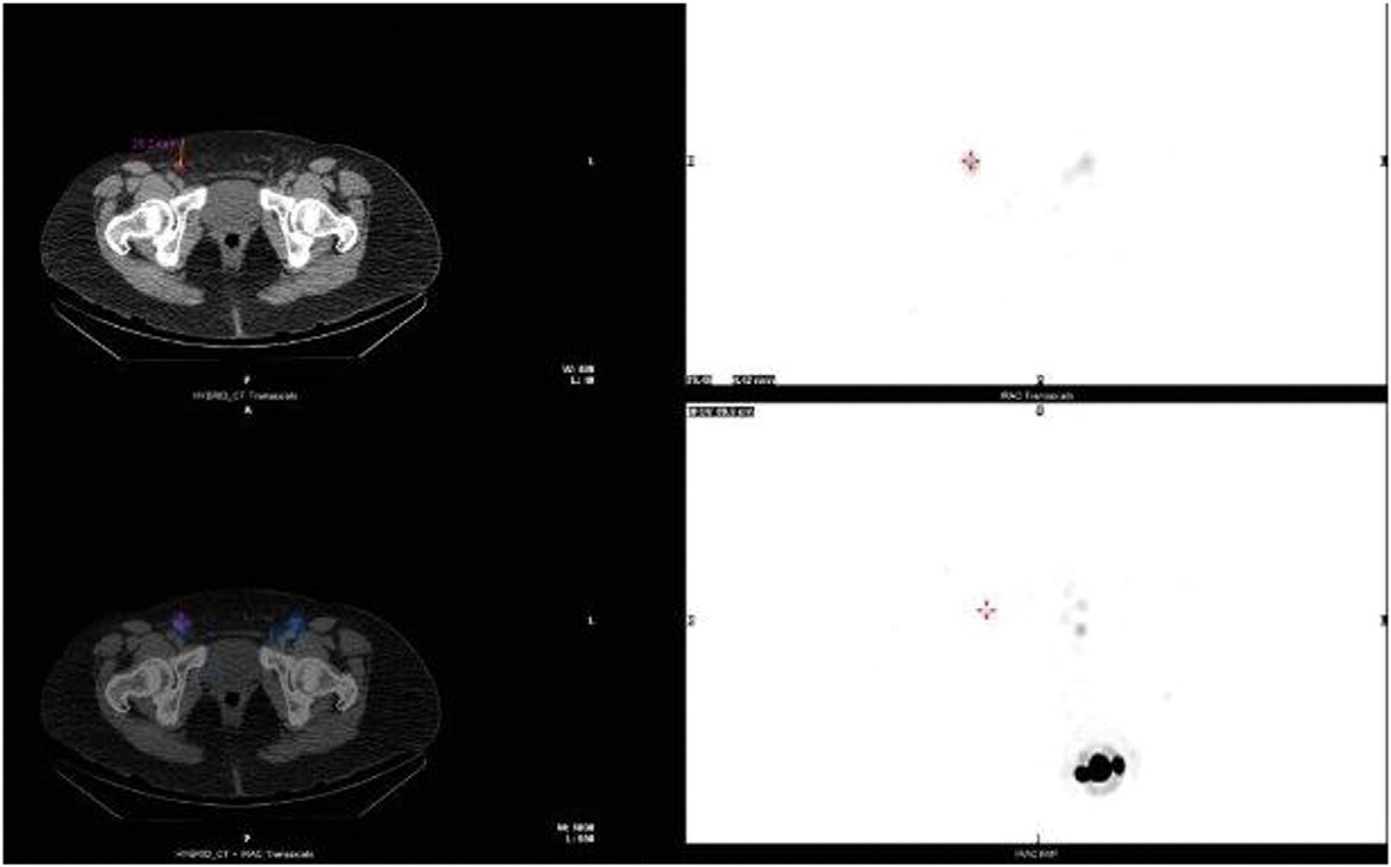
SPECT-CT confirming the presence of a contralateral sentinel lymph node.

Previously published work has suggested value in routinely performing preoperative ultrasound +/- fine needle aspiration cytology of relevant lymph node basins prior to offering a SLNB.^4^ Our protocol for the assessment and management of melanoma patients is based on NICE guidelines and in this case preoperative imagining would only have been performed if metastases were clinically suspected.^5^ It may be true to suggest that ultrasound imaging would have identified the lymph node metastases without the need for the SLNB, however, in line with NICE guidance this is not something routinely offered at our centre. This was also the rationale for not performing a staging CT prior to the SLNB. The SPECT-CT was performed purely as part of the mapping procedure due to the unusual location of the technetium activity. Furthermore, as the lymph nodes showing uptake did not appear overtly pathological on this, there was no indication to perform staging imaging at this point. Whilst the latest NICE guidance does suggest offering staging scans to individuals with stage IIB melanoma, due to significant capacity issues at our centre this is not something that we currently offer.

Variable lymphatic drainage patterns have been described for numerous anatomical locations. The skin of the back has been shown to drain to nodes within the triangular intermuscular space as well as those in the retroperitoneal, intercostal, paravertebral and paraaortic regions.^6^ Drainage from the head and neck region can also be unpredictable, highlighted by Komenaka et al. who reported a case of a facial melanoma demonstrating lymphatic drainage to the contralateral side of the neck.^7^ There is also frequent drainage to interval nodes that lie outside the lymph node basins. In contrast, the lower limb is known to have much more predictable lymphatic drainage pathways. A literature search of MEDLINE and EMBASE identified no documented cases of contralateral sentinel lymph nodes from lower extremity melanomas and so to our knowledge this is the first case of its kind. We do, however, recognise that we cannot say with absolute certainty whether this demonstrates a true anatomical variant or whether the lymphatic drainage had become aberrant due to the metastases. Irrespective of this, our hope is that this case serves as a crucial reminder for clinicians to always examine the contralateral lymph node basin of lower limb melanoma patients.

## Conflict of interest

None.
